# Spondylodiscitis due to *Parvimonas micra* diagnosed by the melting temperature mapping method: a case report

**DOI:** 10.1186/s12879-017-2690-4

**Published:** 2017-08-23

**Authors:** Yoshitsugu Higashi, Shigeki Nakamura, Hideki Niimi, Tomohiro Ueno, Kaoru Matsumoto, Koyomi Kawago, Ippei Sakamaki, Isao Kitajima, Yoshihiro Yamamoto

**Affiliations:** 1 Department of Clinical Infectious Diseases, Graduate School of Medicine and Pharmaceutical Sciences for Research, University of Toyama, Toyama, Japan; 2 Department of Chemotherapy and Mycoses, National Institutes of Infectious Diseases, Tokyo, Japan; 3 Department of Laboratory Medicine, Toyama University Hospital, Toyama, Japan

**Keywords:** Spondylodiscitis, *Parvimonas micra*, Melting temperature mapping method

## Abstract

**Background:**

It has been suggested that more than 100 bacterial species can be identified using only seven universal bacterial primer sets in the melting temperature (Tm) mapping method and that these findings can be obtained within 3 h of sterile site collection.

**Case presentation:**

A 67-year-old Japanese man with type 2 diabetes visited our hospital complaining of progressive lower back pain for 2 months. The patient was suspected to have spondylodiscitis on magnetic resonance imaging of the spine. Blood culture and transcutaneous vertebral biopsy were subsequently performed. Using the Tm mapping method, *Parvimonas micra* was detected from a transcutaneous vertebral biopsy specimen in 3 h. Gram-positive cocci were also detected by Gram staining and *P. micra* was identified directly from the anaerobic blood culture by matrix-assisted laser desorption ionization-time of flight mass spectrometry. Four days after admission, the biopsy specimen culture isolate was identified as *P. micra*.

**Conclusions:**

The Tm mapping method may be useful for the diagnosis of bacterial infections where diagnosis is challenging because of the difficulty of culturing.

## Background

*Parvimonas micra* (*P. micra*) is an anaerobic Gram-positive coccus normally found in the oral cavity, respiratory systems, and gastrointestinal and female genitourinary tracts. Originally known as *Peptostreptococcus micros*, the organism was reassigned to the *Micromonas* genus in 1999, and then reclassified within the *Parvimonas* genus in 2006 [1]. The most common manifestations of *P. micra* infection are periodontal infections and deep organ abscesses [2]. In recent times, *P. micra* has been increasingly isolated or detected, and recognized as the pathogen, in various invasive human infections owing to the widespread use of diagnostic technology such as matrix-assisted laser desorption ionization-time of flight (MALDI-TOF) mass spectrometry and 16S rRNA sequencing [3–9]. However, diagnosis is often challenging due to the difficulty of culturing this anaerobic organism.

Rapid and accurate identification of pathogenic microorganisms from clinical specimens is invaluable for the management of infections. Niimi et al. reported the novel “melting temperature (Tm) mapping method” for rapidly identifying the dominant bacteria in a clinical sample from a sterile site [10]. This study suggested that more than 100 bacterial species can be identified by employing only seven primer sets and that these findings can be obtained within 3 h of sterile site collection. Here, we report a case of spondylodiscitis due to *P. micra,* which was diagnosed by the Tm mapping method.

## Case presentation

**Fig. 1:**
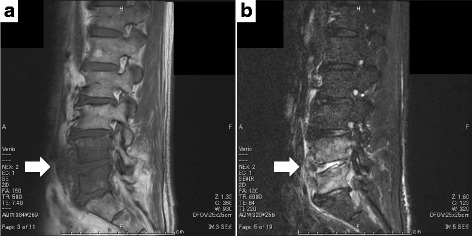
Magnetic resonance imaging of the patient’s spine. Magnetic resonance imaging shows low intensity at the L4 and L5 vertebral bodies and L4–L5 disc space in T1-weighted images (**a**) and hyperintensity in T2-weighted images (**b**) 2 days before admission (*white arrows*)

A 67-year-old Japanese man with type 2 diabetes visited our hospital with complaints of progressive lower back pain for 2 months. He had sensory impairment in the right leg. Cardiovascular, respiratory, and abdominal examinations were unremarkable. His laboratory findings were as follows: white blood cell count, 13,460 cells/μL; hemoglobin level, 16.6 g/dL; platelet count, 39.6 × 104/μL; C-reactive protein level, 3.53 mg/dL; serum total protein level, 7.5 g/dL; albumin level, 2.9 g/dL; lactate dehydrogenase level, 148 IU/L; aspartate aminotransferase level, 29 IU/L; alanine aminotransferase level, 24 IU/L; γ-glutamyltransferase level, 78 IU/L; blood urea nitrogen level, 15 mg/dL; creatinine level, 0.85 mg/dL; and glycated hemoglobin concentration, 7.2%. Magnetic resonance imaging detected low intensity at the L4 and L5 vertebral bodies and L4–L5 disc space in T1-weighted images and hyperintensity in T2-weighted images (Fig. 1). Therefore, the patient was suspected to have spondylodiscitis, and blood culture and transcutaneous vertebral biopsy were performed.

**Fig. 2:**
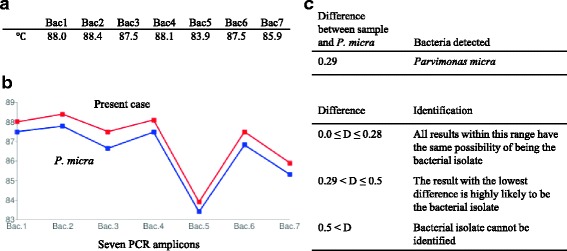
Identification of bacterial pathogen using the melting temperature mapping method. Nested polymerase chain reaction (PCR) of DNA extracted following needle-biopsy sampling of the vertebral disk, using seven universal bacterial primer sets. The melting temperatures (**a**) of the seven bacterial primer sets (Bac1–7) were mapped on dimension leads (**b**). The unique shape of this map matches that of *Parvimonas micra* filed in the online database. The difference between the values for the sample and *P. micra* is 0.29; therefore, the sample is highly likely to contain *P. micra* (**c**) [10]

On the day of admission, Gram staining of a sample taken directly from the anaerobic blood culture bottle showed the presence of Gram-positive cocci; it had taken 66 h for the sample to become blood culture positive. MALDI-TOF mass spectrometry detected *P. micra*, and the patient was suspected to have spondylodiscitis due to *P. micra*. Subsequently, Tm mapping was performed using 1 ml of the remaining needle biopsy sample from the vertebral disk. After DNA was isolated from the pellets using a DNA extraction kit (High Pure PCR Template Preparation Kit, Roche Applied Science, Germany) in accordance with the supplier’s instructions, we conducted nested polymerase chain reaction (PCR) of DNA, using seven universal bacterial primer sets. The data profile was analyzed using the Roter-GeneQ^®^, and the Tm values were identified. The seven Tm values were mapped on two dimensions, and by comparing it to the the database, *P. micra* was identified within 3 h (Fig. 2) [10]. The minimum inhibitory concentrations of antimicrobial agents for *P. micra* isolates from blood cultures were as follows: penicillin G, ≤ 0.03 mg/L; amoxicillin, ≤ 0.03 mg/L; clindamycin, 0.25 mg/L; minocycline, ≤ 0.25 mg/L; cefmetazole, ≤ 1.0 mg/L; and meropenem, ≤ 0.25 mg/L. We initiated intravenous drip infusion of ampicillin/sulbactam at a conventional dose of 3 g/h at 6-h intervals. On day 4 of admission, the biopsy specimen culture revealed the presence of *P. micra*.

Prior to the onset of lower back pain, the patient had refused dental extraction for periodontitis. As this could have been the source of infection, the patient underwent tooth extraction on day 7 of admission. Transthoracic echocardiography did not detect any vascular embolization, indicating the absence of infective endocarditis. After initiating antimicrobial treatment, inflammatory markers including the leukocyte count and serum levels of C-reactive protein gradually improved, and on day 19 of admission, ampicillin/sulbactam administration was replaced by ampicillin (8 g/day) administration. However, because subjective symptoms persisted, the patient underwent L4–S1 decompression and instrumented spinal fusion on day 53 of admission. The patient’s condition improved postoperatively, and we discontinued ampicillin administration on day 72 of admission.

## Discussion and conclusions

Spondylodiscitis in adults is often the result of hematogenous seeding of the adjacent disc space from a distant focus and accounts for approximately 1% of skeletal infections. Mortality due to spondylodiscitis in the antibiotic era is <5%, and the rate of residual neurologic deficits among survivors is <7% [11]. However, delays in diagnosis may cause disabling complications. The rate of diagnosis by vertebral biopsy and blood culture is reported to be only 77 and 58%, respectively [12]. *Staphylococcus aureus* is the most commonly isolated organism; other detected pathogens include *Streptococcus spp.*, *Escherichia coli*, *Pseudomonas aeruginosa*, and *Candida spp*. Spondylodiscitis caused by anaerobic bacteria is relatively rare. However, in some vertebral biopsy-negative or blood culture-negative cases, anaerobic organisms might be the pathogenic organism. Thus, the diagnosis of spondylodiscitis is often challenging due to the difficulty of culturing anaerobic organisms.

*P. micra* is an anaerobic Gram-positive coccus and its diagnosis is often challenging because it is difficult to culture. Furthermore, its pathogenicity has not been studied extensively. A previous study reported that the strong proteolytic activity of *P. micra* could be important in abscess development [13]. *P. micra* forms hydrogen sulfide, which is cytotoxic, from glutathione, a tripeptide involved in intracellular defense against reactive oxygen metabolites [14].

Including this case, a total of 13 cases of spinal infection due to *P. micra* have been previously reported in adults (mean age, 70.1 ± 11.7 years; 7 men) (Table 1) [15–17]. Most of these cases exhibited underlying diseases (12 cases) and/or intraoral defects (8 cases). Furthermore, in almost all cases (12 cases) cultures were positive for *P. micra* (blood, *n* = 6; vertebral biopsy, *n* = 3; surgical specimen, *n* = 3; others, *n* = 3). MALDI-TOF mass spectrometry or 16S rRNA sequencing analysis was required for diagnosis in 8 cases. In 12 of the 13 cases, a single organism was isolated, and only one case report described co-infection with other bacteria including *Fusobacterium nucleatum*.

**Table 1:** Thirteen reported cases of spinal infection due to *Parvimonas micra*

Case	Age	Sex	Underlying disease	Sample	Method for definitive diagnosis	Antimicrobial therapy	Outcome	Year	Reference number
1	70	M	Prostatauxe	Needle-biopsy sampling of vertebral disk	Culture	6 weeks of i.v. CLDM	Recovered	1986	15
2	70	M	Ulcerative colitis	Cerebrospinal fluid specimen	Culture	4 weeks of i.v. penicillin and 4 weeks of oral AMPC in addition to Metronidazole	Recovered	2000	16
3	84	M	Periodontitis	Surgical bone sampling	16S rRNA sequencing	8 weeks i.v. ABPC/SBT and 4 weeks of oral AMPC/CVA	Recovered	2014	3
4	85	F	Periodontitis	Blood culture	Culture	12 days of i.v. Doripenem and 4 weeks of i.v. ABPC, 8 weeks of oral AMPC	Recovered	2014	3
5	49	M	After dental extraction Spinal instrumentation	Surgical bone sampling	Culture, MALDI-TOF mass	6 weeks of i.v. CTRX and oral Metronidazole	Recovered	2015	5
6	72	M	After dental extraction	Needle biopsy sampling of vertebral disk	Culture, 16S rRNA sequencing	6 weeks of i.v. PIPC/TAZ and 2 weeks of oral AMPC/CVA	Recovered	2015	6
7	72	F	Chronic osteoarthritis	Epidural abscess	Culture, MALDI-TOF mass	4 weeks of i.v. PIPC/TAZ	Recovered	2015	6
8	83	M	Ischemic heart disease	Surgical bone sampling and blood culture	Culture, MALDI-TOF mass	15 days of i.v. AMPC + GEM and 12 weeks of oral CLDM + RFP	Recovered	2015	7
9	55	F	After dental treatment	Epidural abscess	Culture and MALDI-TOF mass, 16S rRNA sequencing	6 weeks of i.v. ABPC/SBT and 10 weeks of oral Metronidazole	Recovered	2015	8
10	59	F	Dental caries with an apical granuloma	Blood culture	Culture	14 weeks of oral AMPC	Recovered	2015	17
11	82	F	Dental apical granuloma	Blood culture	Culture	i.v. CTRX + GEM and oral AMPC (total 6 weeks)	Recovered	2015	17
12	60	F	Unknown	Blood culture	Culture	i.v. CTRX + GEM and oral AMPC (total 12 weeks)	Recovered	2015	17
13	67	M	Periodontitis, Diabetes mellitus	Needle biopsy sampling of vertebral disk and blood culture	Culture, MALDI-TOF mass and Tm mapping method	2 weeks of i.v. ABPC/SBT and 8 weeks of i.v. ABPC	Recovered	2015	Present case

*MALDI-TOF mass* Matrix-assisted laser desorption ionization-time of flight mass spectrometry, *Tm* melting temperature, *i.v.* intravenous, *CLDM* clindamycin, *ABPC* ampicillin, *ABPC/SBT* ampicillin/sulbactam, *AMPC* amoxicillin, *AMPC/CVA* amoxicillin/clavulanic acid, *CTRX* ceftriaxone, *PIPC/TAZ* piperacillin/tazobactam, *GEM* gentamicin, *RFP* rifampin

The treatment for spondylodiscitis due to *P. micra* includes antimicrobial therapy with or without surgery. *P. micra* is usually highly susceptible to antibiotics including penicillin, imipenem, clindamycin, metronidazole, and vancomycin, although penicillin- and clindamycin-resistant forms of *P. micra* have been reported [18]. However, the optimal duration of therapy for spondylodiscitis is unknown. Usually, antibiotics are administered intravenously for 4–6 weeks, and most patients are given further oral therapy for 2–6 weeks [12]. Among the spondylodiscitis cases reviewed here, 12 and 5 cases, respectively, involved antibiotic treatment for ≥6 weeks and ≥12 weeks.

Rapid and accurate identification of pathogenic microorganisms from clinical specimens is invaluable for the management of infections. Kumar et al. reported that effective antimicrobial administration within the first hour of documented hypotension was associated with an increased survival to hospital discharge in adult patients with septic shock [19]. However, anaerobic bacteria grow slowly, and therefore, they need a relatively long time for isolation from pure cultures. In a clinical diagnostic microbiology laboratory, the use of new methods of identifying bacterial isolates, such as MALDI-TOF mass spectrometry and 16S rRNA sequencing, is increasing. MALDI-TOF mass spectrometry decreases the time required for identification of the organism by approximately 1 day in clinical microbiology workflows compared to conventional methods [20]. Moreover, this method is reportedly useful for identifying anaerobic bacteria [21]. Although 16S rRNA sequencing allows direct identification from a sample, it has limited applications in the diagnosis of infectious diseases in clinical practice, since gene sequencing analyses tend to be complex, long, and expensive.

In the present case, we detected *P. micra* rapidly and directly from the biopsy specimen within 3 h using the Tm mapping method. Niimi et al. reported that the Tm mapping method was useful for rapid identification of dominant bacteria in a clinical sample collected from a sterile site. Using this method, more than 100 bacterial species could be identified using only seven primer sets within 3 h of sample collection from a sterile site [10]. In addition, the results of the Tm mapping method matched those from culturing in 85% (171 of 200) of patients [10]. Furthermore, 98% (128/130) of samples that tested negative according to the Tm mapping method, were confirmed to be negative using the culturing method. Importantly, all these findings were obtained within a few hours of direct specimen collection. In the present case, the Tm mapping method using needle biopsy sampling of the vertebral disk was useful, but not essential, as the blood culture was herein positive concomitantly.

Unfortunately, bacterial isolation using the Tm mapping method is often challenging in cases of polymicrobial infection. Ultimately, the Tm mapping method may be useful in severe systemic infections such as sepsis shock, and infections of sterile lesions such as spondylodiscitis or meningitis.

To conclude, we describe herein a case of spondylodiscitis due to *P. micra*, which was diagnosed by the Tm mapping method. Owing to the recent widespread use of diagnostic technology, *P. micra* has been isolated more often and identified as the causative pathogen in various invasive human infections. Thus, the Tm mapping method may be useful in diagnosing bacterial infections whose diagnosis is challenging because of a difficulty in culturing.

## Abbreviations

MALDI-TOF: Matrix-assisted laser desorption ionization-time of flight
*P. micra*: *Parvimonas micra*
Tm: Melting temperature

## References

[1] Tindall BJ, Euzeby JP (2006). Proposal of *Parvimonas* gen. Nov. and *Quatrionicoccus* gen. Nov. as replacements for the illegitimate, prokaryotic, generic names *Micromonas* Murdoch and Shah 2000 and *Quadricoccus* Maszenan et al. 2002, respectively. Int J Syst Evol Microbiol.

[2] Murdoch DA (1998). Gram-positive anaerobic cocci. Clin Microbiol Rev.

[3] Uemura H, Hayakwa K, Shimada K, Tojo M, Nagamatsu M, Miyoshi-Akiyama T (2014). *Parvimonas micra* as a causative organism of spondylodiscitis: a report of two cases and a literature review. Int J Infect Dis.

[4] Gomez CA, Gerber DA, Zambrano E, Banaei N, Deresinski S, Blackburn BG (2015). First case of infectious endocarditis caused by *Parvimonas micra*. Anaerobe.

[5] George IA, Pande A, Parsaei S (2015). Delayed infection with *Parbimonas micra* following spinal instrumentation. Anaerobe.

[6] Jones SL, Riordan JW, Glasgow AL, Botes J, Boutlis CS (2015). Two cases of spondylodiscitis caused by *Parvimonas micra*. Intern Med J.

[7] Pilmis B, Israel J, Le Monnier A, Mizrahi A (2015). Spondylodiscitis due to anaerobic bacteria about a case of *Parvimonas micra* infection. Anaerobe.

[8] Endo S, Nemoto T, Yano H, Kakuta R, Kanamori H, Inomata S (2015). First confirmed case of spondylodiscitis with epidural abscess caused by *Parvimonas micra*. J Infect Chemother.

[9] Ko JH, Baek JY, Kang CI, Lee WJ, Lee JY, Cho SY (2015). Bacteremic meningitis caused by *Parvimonas micra* in an immunocompetent host. Anaerobe.

[10] Niimi H, Ueno T, Hayashi S, Abe A, Tsurue T, Mori M (2015). Melting temperature mapping method: a novel method for rapid identification of unknown pathogenic microoganisms within three hours of sample collection. Sci Rep.

[11] Berbari EF, Kanj SS, Kowalski TJ, Darouiche RO, Widmer AF, Schmitt SK (2015). 2015 Infectious Diseases Society of America (IDSA) clinical practice guidelines for the diagnosis and treatment of native vertebral osteomyelitis in adults. Clin Infect Dis.

[12] Mylona E, Samarkos M, Kakalou E, Fanourgiakis P, Skoutelis A (2009). Pyogenic vertebral osteomyelitis: a systematic review of clinical characteristics. Semin Arthritis Rheum.

[13] Murdoch DA, Mitchelmore IJ, Tabaqchali S (1988). *Peptostreptococcus micros* in polymicrobial abscesses. Lancet.

[14] Carlsson J, Larsen JT, Edlund MB (1993). *Peptostreptococcus micros* has a uniquely high capacity to form hydrogen sulfide from glutathione. Oral Microbiol Immunol.

[15] Papasian CJ, McGregor DH, Hodges GR, Kennedy J (1986). Peptostreptococcal vertebral osteomyelitis. J Clin Microbiol.

[16] Leder KS, Barlam TF (2000). A case of paraspinal abscess and diskitis due to *Peptostreptococcus micros*. Clin Infect Dis.

[17] Gahier M, Cozic C, Bourdon S, Guimard T, Cormier G (2015). Spinal infections caused by *Parvimonas micra*. Med Mal Infect.

[18] Brazier J, Chmelar D, Dubreuil L, Feierl G, Hedberg M, Kalenic S (2008). European surveillance study on antimicrobial susceptibility of gram-positive anaerobic cocci. Int J Antimicrob Agents.

[19] Kumar A, Roberts D, Wood KE, Light B, Parrillo JE, Sharma S (2006). Duration of hypotension before initiation of effective antimicrobial therapy is the critical determinant of survival in human septic shock. Crit Care Med.

[20] Huang AM, Newton D, Kunapuli A, Gandhi TN, Washer LL, Isip J (2013). Impact of rapid organism identification via matrix-assisted laser desorption/ionization time-of-flight combined with antimicrobial stewardship team intervention in adult patients with bacteremia and candidemia. Clin Infect Dis.

[21] Nagy E, Becker S, Kostrzewa M, Barta N, Urban E (2012). The value of MALDI-TOF MS for the identification of clinically relevant anaerobic bacteria in routine laboratories. J Med Microbiol.

